# Temporary Transvenous Cardiac Pacing in Patients With Acute Myocardial Infarction Predicts Increased Mortality

**DOI:** 10.4021/cr111w

**Published:** 2012-01-20

**Authors:** Yasir Yaqub, Alejandro Perez-Verdia, Leigh A. Jenkins, Shermila Sehli, Robert L. Paige, Kenneth M. Nugent

**Affiliations:** a Department of Internal Medicine, Texas Tech University Health Science Center, USA; b Department of Mathematics and Statistics, Texas Tech University, Lubbock Texas, USA

**Keywords:** Acute myocardial infarction, Bradyarrhthymias, Temporary pacemakers, Mortality

## Abstract

**Background:**

Temporary pacemakers (TP) are used in emergency situations for severe bradyarrhythmias secondary to acute myocardial infarction (AMI) and to non-AMI related cardiac disorders. TP have been studied previously in AMI patients treated with thrombolytic therapy; limited information is available on current outcomes in AMI patients treated with percutaneous coronary intervention.

**Methods:**

We reviewed the indications, complications, and mortality associated with TP insertion over a four year period (2003 - 2007) at a university hospital.

**Results:**

Seventy-three temporary pacemakers were inserted (47 men, 26 women) during this period. The mean age was 65.2 years. TP were used in 29 AMI patients (39.7 % of total) and 44 non-AMI patients (60.3% of total). The duration of TP use was 2.6 ± 0.4 days in the whole cohort, 2.46 % of all AMI patients (29/1180) admitted during this period required a TP. Six of these patients requiring a TP required a permanent pacemaker. Eight patients with AMI and a TP died (27.6%). In contrast 8.9 % of AMI patients not requiring a TP died (P < 0.01). There were no statistically significant differences between the AMI and non-AMI groups in the duration of temporary pacing (2.4 ± 0.6 days vs. 2.8 ± 0.4 days), in complications (27.6% vs. 29.5%), or in mortality (27.6% vs. 15.9%). The need for a permanent pacemaker (PPM) differed significantly between the AMI and non-AMI patients (20.7% vs. 54.5%; P < 0.05).

**Conclusion:**

Our results indicate that AMI patients infrequently require a TP and that approximately 20% of these patients require a PPM. These results suggest that early revascularization of the conduction system with current interventional techniques has decreased the need for TP in AMI patients. However, this group requires more intensive monitoring as the mortality rate in this group of patients is significantly higher than the other AMI patients not requiring TP.

## Introduction

Current ACC/AHA indications for temporary artificial pacing include severe bradyarrhythmias (high grade 2nd or 3rd degree atrioventricular block (AVB] or severe symptomatic bradycardia) and bradyarrhythmias complicating acute myocardial infarction (AMI) if excessively slow escape ventricular dysrhythmia, hypotension, and/or hypoperfusion unresponsive to atropine occur. Isolated left anterior fascicular block or new bundle branch block without AV block are not indications for pacing [1]. The cardiac conduction system has a rich blood supply, and AMI, especially in inferior and anterior wall locations, leads to ischemia of the conducting system [2, 3]. Infarct size correlates with high grade AV block [4-6] and the need for temporary and permanent pacemakers. Hence, the use of temporary pacemaker support in AMI patients potentially reflects the extent of injury and predicts short term prognosis based on electrocardiogram (ECG) features, infarct location and Killip class [1-23].

The effect of revascularization by thrombolytics on the conduction system has been reported [2, 4, 6, 7, 9, 10, 13]. However, there is little information available on the need for temporary or permanent pacemakers in AMI patients managed with percutaneous coronary intervention (PCI). Advances in PCI techniques and other concurrent changes in treatment strategies could have changed the frequency and characteristics of complete or high grade AV block in patients admitted with AMI. We wanted to determine the clinical characteristics and short term outcomes of patients with acute MI who required a temporary pacemaker at our hospital. We compared this group of patients with other patients requiring a TP and with all patients admitted with an AMI.

## Methods

### Study design

This retrospective medical record review was conducted at a university-based hospital with a cardiology fellowship training program and had the approval of the Texas Tech University Health Sciences Center Institutional Review Board. The patients were identified with billing codes for temporary transvenous pacemaker insertion at our institution between July 2003 and June 2007. Clinical information was collected with standardized data abstraction forms/Microsoft Excel sheets by a physician reviewer. Uncertainties about diagnosis, ECG, or complications were referred to the attending cardiologist for clarification. We also obtained information (mortality and length of stay) on all patients admitted with an AMI between July 2003 and June 2007 using ICD-9 diagnosis and procedure codes.

### Selection and description of patients

All medical records having a procedure code for temporary transvenous pacemaker insertion between July 2003 and June 2007 were screened for enrollment. Records were eligible for inclusion if a temporary transvenous pacemaker wire was placed in the cardiac catheterization laboratory, critical care setting, or emergency center by a cardiology faculty member or fellow. Information on patient characteristics, temporary transvenous pacemaker indication, insertion approach used, and complications was obtained from the medical record review. All medical records were available in electronic and paper formats and included formal procedure notes. The records were reviewed from time of admission to discharge or death in hospital to identify immediate and delayed complications arising from the temporary transvenous pacemaker insertion. Pacemaker malfunction was defined as failure of capture, or sense, or both. Only complications directly attributed to the temporary pacemaker were reported. These included transient arrhythmias (ventricular tachycardia/fibrillation, PVC’s) and pacemaker malfunction intra- and post-operatively. The primary cause for mortality was determined by primary discharge diagnosis.

### Temporary pacemaker placement

Temporary pacemaker electrodes were placed in the cardiac catheterization lab using fluoroscopic guidance or in the coronary care unit. Electrode catheters were placed either by a cardiologist or by a cardiology fellow under the supervision of a cardiologist faculty. Our hospital uses a 110 cm fluoroscopically guided pacing catheter for temporary transvenous pacing (Pacel, 5F, 110 cm). For unguided catheters, St. Jude Medical Pacel flow directed pacing catheter is used (5 F, 110 cm). Medtronic 5388 temporary dual chamber pacemakers were utilized in most of cases. Guidance was attained by using continuous ECG monitoring. A pacing threshold of < 1.0 mA was considered adequate. Initial output was set at twice the diastolic pacing threshold. Electrode positioning was confirmed by chest x-ray in all cases by a portable x-ray in the coronary care unit. Continuous electrocardiographic monitoring (telemetry) and complete bed rest were required until a permanent pacemaker was implanted or the symptoms causing the indication for a temporary pacemaker had resolved.

### Statistical analysis

SPSS^®^ software v.11.0 for Windows and Megastat Microsoft Excel software were used in the statistical analyses. Results were expressed as mean ± standard deviation (SD) for continuous variables. Student’s t test was used for independent samples with quantitative variables; the Chi square and the Fisher exact tests were used for comparison of qualitative variables. Significance was set at a P-value < 0.05.

## Results

Seventy-three patients had a TP placed during this time period. The mean age was 65.2 years (range 21 - 94); 47 were men and 26 were women. Important clinical variables and demographic features are listed in Table 1. All indications are listed in Table 2. The TP was inserted through the femoral vein in 90% of the cases; the subclavian vein in 6%, and the jugular vein in 4%. The mean ventricular rate at the time of TP placement was 35 ± 8 beats per minute. The mean duration of TP use was 2.6 ± 0.4 days. The length of hospitalization correlated with the number of days of TP insertion (r = 0.432, P < 0.01). Thirty permanent pacemakers (41.1% of patients), including implantable cardioverter-defibrillators, were placed after temporary pacemaker insertion.

**Table 1:** Demographic Characteristics of the Patients

Age	65.2 ± 14.8 years (range 21-94)
Sex	47 (64%) Male, 26 (36%) Females
Comorbid conditions	Number of patients
Coronary Artery Disease	31
Diabetes Mellitus	27
Hypertension	48
Peripheral Vascular Disease	2
Dyslipidemia	12
Sick Sinus Syndrome	12
Acute Kidney Insufficiency	6
Chronic Kidney Disease	3
Overdose	4
Trauma	1
Meningitis	1

**Table 2:** Temporary Pacemaker Indications

Indications	Patient number
Acute Myocardial Infarction Group	
Bradyarrhythmias including complete heart block from AV nodal artery involvement requiring TP	29
Non- Acute Myocardial Infarction Group	
Complete Heart Block from Age related fibrosis-Lenegre’s disease, Cardiomyopathy, etc.	14
2nd degree AV Block ( symptomatic type 2)	2
Trifascicular Block	1
Severe Bradycardia from CNS/PNS events	3
Sinus Nodal Dysfunction/SSS	5
Secondary 2nd degree AV blocks/ complete AV block	
1)Electrolyte abnormalities and renal failure	4
2)Drug toxicity	4
Malfunction/ Infection of cardiac pacemaker (twiddler syndrome)	4
Miscellaneous (Asystole-4/ Trauma related-1/UGIB-1/Sepsis-1)	7
Total Patients	73

*Non: AMI Complete AV block (Age related fibrosis-Lenegre’s disease, cardiomyopathy etc.); SSS: Sick sinus syndrome; UGIB: Upper GI bleed.

Twenty-nine patients managed with a TP had an AMI (mean age 66.6 ± 10.4 years). Eight patients (28%) presented with inferolateral MI, seven patients with (24%) inferoposterior MI, nine patients (31%) with NSTEMI, two patients with lateral wall MI and three patients with anterior wall MI. All had an elevated troponin T level. Twenty-two patients had angiography with percutaneous intervention with a drug eluting stent. Four patients with NSTEMI and three patients with STEMI had angiography done without attempted PCI either secondary to triple vessel disease requiring ACB or to presentation in cardiac arrest with pulseless electrical activity and severe hemodynamic instability with unsuccessful resuscitation and/or PCI. The mean door to PCI time in STEMI patients was 76.8 minutes (range: 50 -140 minutes). Six patients in the AMI (6/29; 20.7%) required a permanent pacemaker, including four patient with a STEMI and two patients with a NSTEMI. Twenty-four patients (24/44, 54.5%) with a non-AMI diagnosis required a PPM (P < 0.05 in comparison with AMI patients). Eight patients with AMI had complications. These included episodes of non-sustained ventricular tachycardia in six patients and junctional rhythms and bradyarrhythmias in four patients. Thirteen patients with a non-AMI diagnosis had a complication (P > 0.05 in comparison with AMI patients). Risk factor comparison and outcomes between AMI and non-AMI group are shown in Table 3 and Table 4. Deep venous thrombosis did not develop despite predominant use of the femoral approach.

**Table 3:** Risk Factor Comparison of AMI and Non AMI Groups

	AMI group	Non-AMI Group
Number of patients	29 (39.7 %)	44 (60.3%)
Age	66.6 ± 10.4	64.5 ± 17.1
Male sex	20 (68.9%)	27 (61.4%)
Diabetes Mellitus	13 (44.8%)	14 (31.8%)
Hypertension	24 (82.8%)	24 (54.5 %)
Dyslipidemia	10 (34.5%)	2 (4.5 %)
History of CAD	20 (69%)	11 (25%)
PVD	1 (3.4 %)	1 (2.3%)
CVA	1 (3.4 %)	7 (15.9 %)
Hypothyroidism	1 (3.4 %)	5 (11.5%)

PVD: Peripheral vascular disease; CVA: Cerebrovascular accident; CAD: Coronary artery disease.

**Table 4:** Comparison of AMI and Non AMI Groups

	AMI group	Non-AMI Group	P value
Number of patients	29	44	
Mortality	27.6%	15.9%	P = 0.25
Duration of TP	2.4 days	2.8 days	P = 0.61
Complete AVB	23/29	14/44	P = 0.03
PPM	6/29	24/44	P < 0.05
TP Repositioned	0/29	6/44	P = 0.052
V-Tach episodes	6/29	5/44	P = 0.35
Total complications	8/29	13/44	P = 0.8
Frequent PVC’s	3/29	5/44	P = 0.9

TP: Temporary pacemaker; PPM: Permanent pacemaker; AVB: Atrioventricular block; V-Tach: Ventricular tachycardia.

During this study period 1180 patients had an AMI discharge diagnosis. Therefore, 2.46 % required a TP. The overall mortality was 8.9% (103/1151) in AMI patients not requiring a TP. The mortality rate in the patients with AMI requiring TP was 27.6% (8/29, P < 0.01). Three patients presented in cardiac arrest with PEA and died during attempted PCI. Two patients died of extensive RV infarction, one patient died from acute tamponade accompanying RV infarction, and remaining two patients died before ACB could be attempted. The mortality in the non-AMI patients was 15.9%. No death was attributed to temporary pacemaker insertion or any related complications.

## Discussion

The temporary cardiac pacing may be required in acute myocardial infarction setting [1-23] especially in patients with high grade AV blocks [1]. The atrioventricular (AV) node and the infranodal conduction system have a very rich vascular supply from the atrioventricular nodal artery and collateral circulation [2, 3, 5, 6]. High degree AV blocks occurred in AMI secondary to extensive infarcts [1, 2, 5, 6, 13, 15]. In addition, metabolic phenomena, such as the release of adenosine or potassium, and persistent ischemia of the node and conduction tissue also contribute to AVB [15, 22]. Prior studies have demonstrated that the relevant infarct-related artery was occluded in 83% of patients with AMI and heart blocks [22]. This established the fact that ischemia frequently contributes to the development of high grade blocks in AMI patients and that therapy should focus on this process [6, 15, 22, 23]. Thrombolytic therapy reduces the incidence of complete AVB and improves the prognosis of patients with AMI [4-7, 9, 12, 13]. However, little information is available on the incidence, duration, and need for temporary and permanent pacing in AMI patients with AVB managed with PCI techniques, and questions about these issues led to our review.

Approximately 3% of patients with an AMI our study required TP (29/1180, 2.5%). In our study four patient in our STEMI group (4/19) with acute intervention required PPM. The other two permanent pacemakers in the AMI group were placed in NSTEMI patients who had multi-vessel diseases with 70 - 100% RCA occlusion. Some literature reports indicate that a higher percentage of patients with an AMI require a PPM. For example, Murphy reported that permanent pacemakers were required in 50% of his AMI patients, and Pinneri reported that more than 40% of PPM in his institution were inserted in AMI patients [5, 21]. Our outcomes suggest that early intervention with PCI leads to improved revascularization of the infarct-related artery and associated conduction system and reduces the need for permanent pacemakers in AMI patients requiring temporary pacing. Of course, this study design cannot definitively demonstrate this conclusion.

The mortality in AMI patients requiring a TP was 27.6 % (8/29). This rate is significantly higher than the mortality rate in AMI patients not requiring a pacemaker (103/1151, 8.9%). Mortality in AMI group requiring TP was comparable to our non-AMI patients presenting with diverse clinical syndromes and to the 28% reported in an earlier study [5]. Our complication rates were similar in both patient groups and to those reported in earlier studies. No deaths were attributed to pacemaker-related complications. Therefore, patients with AMI requiring TP are at increased risk for mortality, since high grade AV blocks reflect extensive ischemia and/or infarction [6, 13, 15]. Therefore, the need for TP in AMI patients is a good indicator for disease severity and patient acuity.

Temporary pacing has been associated with multiple complications; the frequency of complications ranges from 13.7% to 33% of patients in literature series [5, 9]. We had 21 complications in 73 patients (30 %). Failure to capture by the temporary pacemaker occurred in 9.6% in our study and in 37% to 43% in other studies [11, 12]. Ventricular tachycardia/fibrillation post insertion occurred in 15% of patients compared to 10% to 37.5% in other literature series [8, 11, 12]. Lethal complications reported with temporary pacemaker insertion, such as cardiac tamponade and infectious complications, did not occur in our study [11, 12, 14]. The route of insertion was mainly the femoral vein (90%) compared to predominance of internal jugular and subclavian routes in previous studies [5, 7, 9, 16, 18], but we did not detect any DVTs post procedure.

Our study has definite limitations. First, this information comes from one hospital and may not be generalizable. Second, known and unknown confounding variables often occur in retrospective studies. We tried to minimize this by identifying all the patients with TP during this period for review and by accounting for as many variables as possible. Third, our study did not take into account the use of trans-cutaneous pacing in our institute, and some of the patients in our cohort did receive trans-cutaneous pacing before TP insertion. The effect, if any, of trans-cutaneous pacemakers would require a separate study with many more patients. Fourth, we did not compare our study results with historical data from our hospital during the period with more frequent thrombolytic therapy, but we did compare it with all AMI patients admitted during this period and with literature reports.

### Conclusion

Multiple factors potentially influence the need for temporary and permanent pacemakers. Therefore, comparing results in different studies with different intervention strategies is difficult. The AMI patients in our hospital who required a TP had an increased mortality rate. This probably reflects the extent of myocardial injury and the complexity of the clinical events associated with AMI. These patients need comprehensive management protocols during the initial phase of hospitalization. Quality improvement projects and registries involving AMI patients requiring TP might improve outcomes and help clarify the causes of death along with long term outcomes in these patients.
